# Prognostic value of the dynamic hepatorenal function on intermediate‐term mortality in TAVI patients with survival to discharge

**DOI:** 10.1002/clc.23940

**Published:** 2022-11-30

**Authors:** Yijun Yao, Jingjing He, Tianyuan Xiong, Fei Chen, Yuanweixiang Ou, Yiming Li, Qi Liu, Zhongkai Zhu, Yi Zhang, Haoran Yang, Yujia Liang, Xin Wei, Xi Li, Yong Peng, Jiafu Wei, Sen He, Qiao Li, Yong Chen, Wei Meng, Guo Chen, Wenxia Zhou, Mingxia Zheng, Xuan Zhou, Mao Chen, Yuan Feng

**Affiliations:** ^1^ Department of Cardiology, West China Hospital Sichuan University Chengdu China; ^2^ Department of Cardiology, Section of Cardiac Ultrasound West China Hospital, Sichuan University Chengdu China; ^3^ Department of Cardiac Surgery, West China Hospital Sichuan University Chengdu China; ^4^ Department of Anesthesiology, West China Hospital Sichuan University Chengdu China; ^5^ Department of Radiology, West China Hospital Sichuan University Chengdu China

**Keywords:** aortic stenosis, hepatorenal function, transcatheter aortic valve implantation

## Abstract

**Background:**

Renal and liver dysfunctions are risk factors for mortality in patients with severe aortic stenosis (AS). Transcatheter aortic valve implantation (TAVI) has the potential to break the vicious cycle between AS and hepatorenal dysfunction by relieving aortic valve obstruction.

**Hypothesis:**

A part of patients can derive hepatorenal function improvement from TAVI, and this noncardiac benefit improves the intermediate‐term outcomes.

**Methods:**

We developed this retrospective cohort study in 439 consecutive patients undergoing TAVI and described the dynamic hepatorenal function assessed by model for end‐stage liver disease model for end‐stage liver disease (MELD)‐XI score in subgroups. The endpoint was 2‐year all‐cause mortality.

**Results:**

Receiver‐operating characteristic analysis showed that the baseline MELD‐XI score of 10.71 was the cutoff point. A high MELD‐XI score (>10.71) at baseline was an independent predictor of the 2‐year mortality hazard ratio (HR: 2.65 [1.29–5.47], *p* = .008). After TAVI, patients with irreversible high MELD‐XI scores had a higher risk of 2‐year mortality than patients who improved from high to low MELD‐XI scores (HR: 2.50 [1.06–5.91], *p* = .03). Factors associated with reversible MELD‐XI scores improvement were low baseline MELD‐XI scores (≤12.00, odds ratio [OR]: 2.02 [1.04–3.94], *p* = .04), high aortic valve peak velocity (≥5 m/s, OR: 2.17 [1.11–4.24], *p* = .02), and low body mass index (≤25 kg/m^2^, OR: 2.73 [1.25–5.98], *p* = .01).

**Conclusion:**

High MELD‐XI score at baseline is an independent predictor for 2‐year mortality. Patients with hepatorenal function improvement after TAVI have better outcomes. For patients with irreversible hepatorenal dysfunction after TAVI, further optimization of the subsequent treatment after TAVI is needed to improve the outcomes.

## 1 INTRODUCTION

There is a vicious cycle between aortic stenosis (AS) and hepatorenal dysfunction.^1^, ^2^, ^3^ Transcatheter aortic valve implantation (TAVI) is currently the guideline‐recommended therapy for patients with severe AS,^4^, ^5^ and it has the potential to break this vicious cycle by relieving aortic valve obstruction. Prior studies showed that TAVI can bring about improvements in renal function^6^ but may also be complicated by kidney injury which is a risk factor for prognosis.^7^, ^8^, ^9^ However, liver function is underappreciated, with a few studies focusing on liver function only in patients with pre‐existing liver diseases.^10^, ^11^, ^12^, ^13^ Despite many studies emphasizing the prognostic value of the combination of renal and liver function on varied types of cardiac pathologies,^14^, ^15^, ^16^, ^17^, ^18^, ^19^, ^20^ there is a paucity of data on TAVI. Moreover, it remains to be seen which part of patients are more likely to derive hepatorenal function improvement from TAVI, and how this benefit influences intermediate‐term mortality. Therefore, we aim to examine the association between hepatorenal function and 2‐year mortality of TAVI and describe the dynamic hepatorenal function changes at baseline, at discharge, and during follow‐up, to identify the effect of TAVI on hepatorenal function in subgroups.

## 2 METHODS

### 2.1 Study design and population

This study was a retrospective cohort analysis. The cohort included consecutive patients who underwent their first TAVI procedure at our institution between January 1, 2013 and March 19, 2019. Patients who died before discharge, underwent redo‐TAVI, with missing serum creatinine or total bilirubin data at baseline or discharge, with an end‐stage renal disease requiring dialysis, or with known liver disease caused by non‐cardiogenic factors (e.g., viral hepatitis, drug use, alcohol use, autoimmune disease, and metabolic disease), with bilirubin elevations from nonhepatic causes (e.g., hemolytic diseases, hereditary hyperbilirubinemia syndrome, Mirrizzi syndrome) were excluded. The patient selection flow diagram is shown in Supporting Information: Figure 1. The etiology of liver dysfunction was confirmed by the diagnosis from medical records, which were drawn based on the proper examination. All patients had routinely undergone abdominal enhanced computed tomography, biochemical, and hepatitis virus testing before TAVI to preliminarily assess the liver condition, and liver biopsy was performed when necessary.

For monitoring, patients routinely received blood sample tests during admission, which included serum creatinine and total bilirubin value. The earliest pre‐TAVI serum creatinine and total bilirubin value measured during hospitalization were used as the baseline lab values. The latest creatinine and total bilirubin value measured before discharge was used as the discharge lab values. The baseline characteristics were obtained from the medical records. The transthoracic echocardiography was performed before and after TAVI during the admission. All echocardiograms were analyzed at an independent echocardiography core laboratory that followed the American Society of Echocardiography/European Association of Cardiovascular Imaging standards for echocardiography core laboratories.^21^ The follow‐ups were carried out at the 1, 3, 6, 12 months, 1 year, and 2 years after TAVI, and the events of death were recorded. The latest serum creatinine or total bilirubin value during the 2‐year follow‐up was defined as the follow‐up value.

The study objectives were as follows: First, to explore the relationship between hepatorenal function at baseline and 2‐year all‐cause mortality after TAVI, and to identify the risk factors associated with hepatorenal dysfunction at baseline. Second, to evaluate short‐term changes in hepatorenal function after TAVI at the point of discharge, and its relationship with 2‐year all‐cause mortality after TAVI, as well as to identify the clinical factors associated with reversible hepatorenal dysfunction. For these two objectives, we analyzed the data of all patients who met the study inclusion criteria (Cohort 1). Third, to observe the changing trend of hepatorenal function improved after TAVI during follow‐up. For this objective, we analyzed the data of patients who had hepatorenal function assessment during follow‐up (Cohort 2).

### 2.2 Assessment of hepatorenal function

As a considerable proportion of patients undergoing TAVI were on vitamin K antagonists, we chose the model for end‐stage liver disease excluding the international normalized ratio (INR) model for end‐stage liver disease (MELD)‐XI score as the measure of hepatorenal function, which does not take into account the INR value. MELD‐XI score was calculated as: 5.11 × ln(serum total bilirubin in mg/dl) + 11.76 × ln(serum creatinine in mg/dl) + 9.44. If a patient had creatinine or total bilirubin lower than 1.0 mg/dl, the logarithm of the value is negative. Therefore, following the customary practice of the United Network for Organ Sharing, the creatinine or total bilirubin lower than 1.0 mg/dl is set to 1.0 mg/dl to prevent negative values.^22^ As a result, if both values are lower than 1.0 mg/dl, the MELD‐XI score is the lowest value and equals 9.44.

### 2.3 Statistical analysis

Statistical analyses were performed using SPSS version 26.0 (SPSS Inc.) and R software (R Foundation). Data were presented as mean ± SD, median (interquartile range), counts (%), or estimates (95% confidence interval). The integrated discrimination improvement (IDI) was used to compare the performance with prediction models by creatinine and by MELD‐XI score. The optimal cutoff value of the MELD‐XI score for the prediction of 2‐year all‐cause mortality was determined by receiver‐operating characteristic (ROC) curve analysis and the Youden index. We defined the MELD‐XI score higher than the cutoff value as the high MELD‐XI score, and those lower than or equal to the cutoff value as the low MELD‐XI score. Baseline characteristics were compared between patients with high and low MELD‐XI scores using Pearson *χ* ^2^ tests for categorical variables and independent sample *t*‐tests for continuous variables. Cox proportional hazards models were used to assess the association between the MELD‐XI score at baseline and 2‐year all‐cause mortality. A univariate Cox model was developed using all preoperative variables in the baseline table, then the variables with *p* < .05 were incorporated into a multivariate Cox model using forward stepwise selection, and *p* < .05 was required to remain in the models. Time‐to‐event analyses were performed using the Kaplan–Meier method, comparing the 2‐year all‐cause mortality hazard ratio (HR) between high and low MELD‐XI score groups.

## 3 RESULTS

### 3.1 Baseline characteristics

A total of 439 patients undergoing TAVI from January 1, 2013 to March 19, 2019 were included in the study (Supporting Information: Figure 2), with 263 patients (59.9%) treated from 2017 to 2019, 176 patients (40.1%) treated from 2013 to 2016. Baseline clinical characteristics are shown in Table 1. Patients were 74 ± 6 years old, with a median Society of Thoracic Surgeons (STS) score of 7.6%. The median length of admission was 7 days. The mortality during the 2‐year follow‐up was 8.9%.

**Table 1:** Baseline characteristics (*n* = 439)

Demographics	
Age, years	74 ± 6
Male	246 (56.0)
Body mass index, kg/m^2^	22.1 ± 3.7
STS score, %	7.6 (4.6–9.1)
STS score ≥8%	184 (41.9)
Symptoms	
Syncope	52 (11.8)
Chest pain	127 (28.9)
Dyspnea	395 (90.0)
NYHA III or IV	389 (87.9)
Cardiovascular conditions	
Hypertension	188 (42.8)
History of atrial arrhythmia	70 (15.9)
Coronary artery disease	168 (38.3)
Previous MI	8 (1.8)
Previous PCI	39 (8.9)
Peripheral arterial disease	201 (45.8)
Prior stroke or TIA	97 (22.1)
Noncardiac conditions	
COPD	225 (58.1)
Diabetes mellitus	83 (18.9)
Echocardiography	
LVEF, %	59 (43–67)
LVEF < 35%	55 (12.5)
Aortic valve mean gradient, mmHg	60 (47–73)
Aortic valve peak velocity, m/s	4.8 (4.3–5.4)
≥Moderate AR severity	127 (28.9)
≥Moderate MR severity	62 (14.1)
≥Moderate TR severity	62 (14.1)
IVS, mm	14 ± 2
Year of procedure	
2013–2016	176 (40.1%)
2017–2019	263 (59.9%)
Procedural and postprocedural data	
Self‐expandable valve	413 (94.1)
Aortic valve mean gradient after TAVI	11 (8–16)
Aortic valve peak velocity after TAVI	2.3 (2.0–2.6)
≥Moderate AR severity after TAVI	5 (1.1)
≥Moderate MR severity after TAVI	24 (5.5)
≥Moderate TR severity after TAVI	25 (5.7)
Bleeding	35 (8.0)
Vascular complication	30 (6.8)
LVEF after TAVI, %	60 (48–65)
LVEF after TAVI < 35%	33 (7.5)
Length of admission, days	9 (7–11)

*Note*: Values are mean ± SD or *n* (%).

Abbreviations: AR, aortic regurgitation; COPD, chronic obstructive pulmonary disease; LVEF, left ventricular ejection fractions; MI, myocardial infarction; MR, mitral regurgitation; NYHA, New York Heart Association; PCI, percutaneous coronary intervention; STS, Society of Thoracic Surgeons; TAVI, transcatheter aortic valve implantation; TIA, transient ischemia attack; TR, tricuspid regurgitation.

The median creatinine was 0.96 mg/dl (interquartile range: 0.81–1.20 mg/dl), and 204 (46.5%) patients had high creatinine (≥1 mg/dl). The median total bilirubin was 0.73 mg/dl (interquartile range: 0.53–1.00 mg/dl), and 107 (24.4%) patients had high total bilirubin (≥1 mg/dl). The median MELD‐XI score at baseline was 9.94 (interquartile range: 9.44–12.12).

### 3.2 Prognostic value of the hepatorenal function at baseline

**Figure 1:**
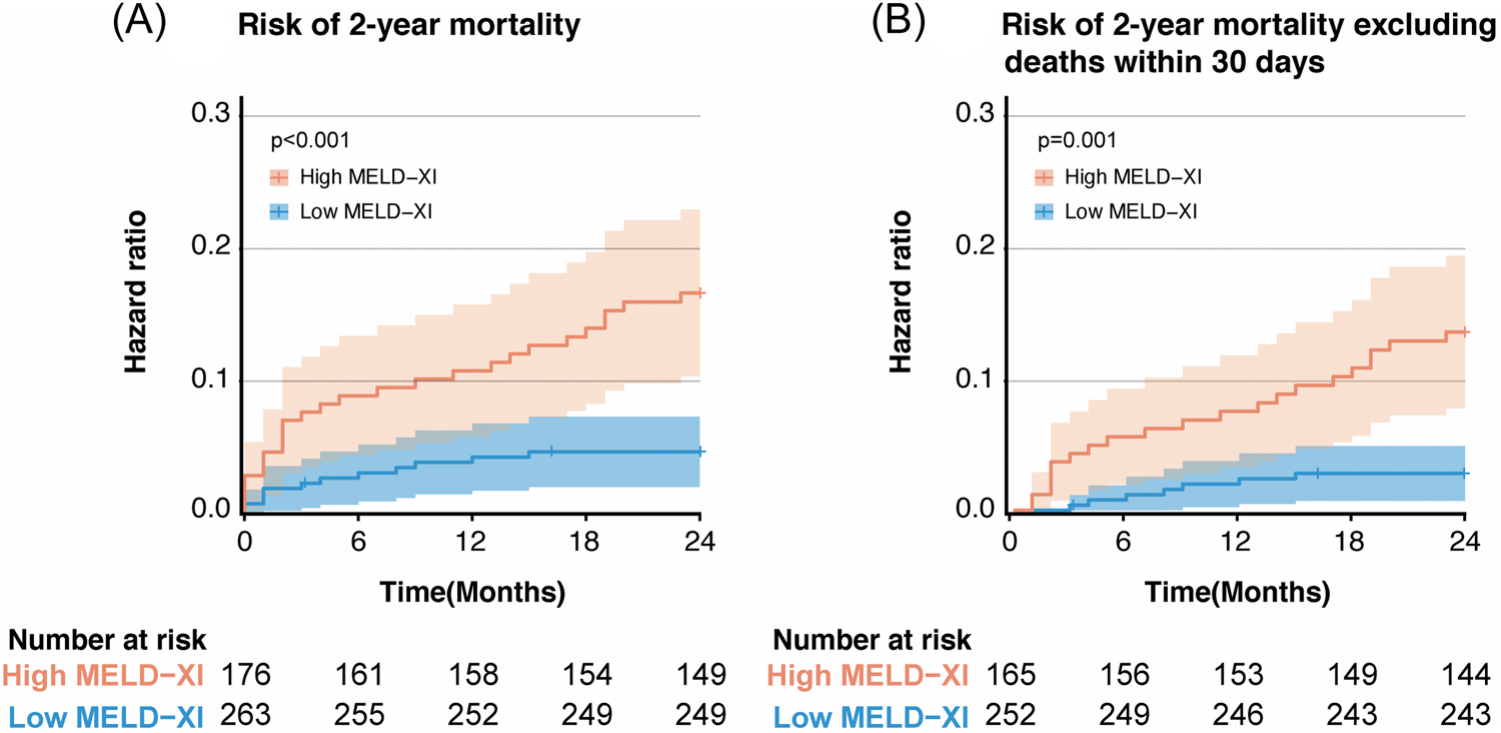
Kaplan–Meier curves for 2‐year all‐cause mortality according to MELD‐XI score. MELD, model for end‐stage liver disease.

Both creatinine (HR: 1.64 [1.17–2.31], *p* = .004) and total bilirubin (HR: 1.69 [1.22–2.35], *p* = .02) at baseline were associated with 2‐year mortality on univariate analysis. MELD‐XI of 10.71 at baseline provided the optimal cutoff point to detect patients at risk for 2‐year mortality according to the ROC analysis. Using 10.71 as the cutoff point, 263(59.9%) patients with low MELD‐XI scores (≤10.71) had 2‐year mortality of 4.6%, and 176 (40.1%) patients with high MELD‐XI scores (>10.71) had 2‐year mortality of 15.3%. A high MELD‐XI score was associated with a higher risk of 2‐year mortality (HR, 3.52 [1.79–6.95], *p* < .001) on univariate analysis (Figure 1A), and still remained as an independent risk factor on multivariate analysis (HR, 2.65 [2.04–11.28], *p* < .001) (Table 2). After excluding the deaths within 30 days, a high MELD‐XI score was still an independent risk factor of higher risk of 2‐year mortality (HR: 4.48 [1.88–10.65], *p* = .001) on multivariate analysis (Figure 1B). Using multivariate regression analysis, tricuspid regurgitation (≥moderate) had the strongest correlation with the MELD‐XI score (standardized *b* = 0.20), followed by mitral regurgitation (≥moderate), diabetes mellitus, aortic valve peak velocity, and STS score (Supporting Information:  Table 1).

**Table 2:** Multivariate model showing risk factors for 2‐year mortality

	HR (95% CI)	*p*
High baseline MELD‐XI score	2.65 (1.29–5.47)	.008
STS score, per %	1.07 (1.02–1.12)	.004
≥Moderate AR severity	0.28 (0.10–0.80)	.02

Abbreviations: AR, aortic regurgitation; CI, confidence interval; HR, hazard ratio; MELD,  model for end‐stage liver disease; STS, Society of Thoracic Surgeons.

The IDI analysis showed a better performance of prediction in the MELD‐XI score than creatinine alone (*p* = .01) (Supporting Information: Figure 2). When combining the STS score with total bilirubin at baseline, it showed better performance in predicting peri‐TAVI mortality (*p* = .02) and intermediate‐term mortality risk (*p* = .04) than the STS score alone (Supporting Information: Figure 3).

### 3.3 Hepatorenal function at discharge

At discharge, the creatinine was 0.88 mg/dl (interquartile range: 0.74–1.06 mg/dl), with a mean reduction of 0.11 mg/dl to that at baseline (*p* < .001). The total bilirubin was 0.74 mg/dl (interquartile range: 0.55–1.02 mg/dl). For the 110 patients with high total bilirubin (≥1 mg/dl) at baseline, the total bilirubin had a mean reduction of 0.28 mg/dl (*p* < .001) at discharge, and 50 (45.5%) improved to the normal level. The MELD‐XI score was 9.44 (interquartile range: 9.44–11.50), with a mean reduction of 0.43 (*p* = .004).

Using 10.71 as the cutoff point, 295 patients with high MELD‐XI scores at discharge had increased risk in 2‐year mortality (HR: 2.84 [1.59–5.09], *p* < .001). High MELD‐XI score at discharge was associated with high MELD‐XI score at baseline (OR: 4.97 [3.09–7.99], *p* < .001), tricuspid regurgitation (≥moderate) after TAVI (OR: 4.67 [1.76–12.42], *p* < .001), chronic obstructive pulmonary disease (odds ratio [OR]: 1.71 [1.05–2.80], *p* = .02), and body mass index (BMI; OR: 1.08 [1.01–1.16], *p* = .03) on multivariable analysis.

### 3.4 Factors and prognostic value of hepatorenal function changes

**Figure 2:**
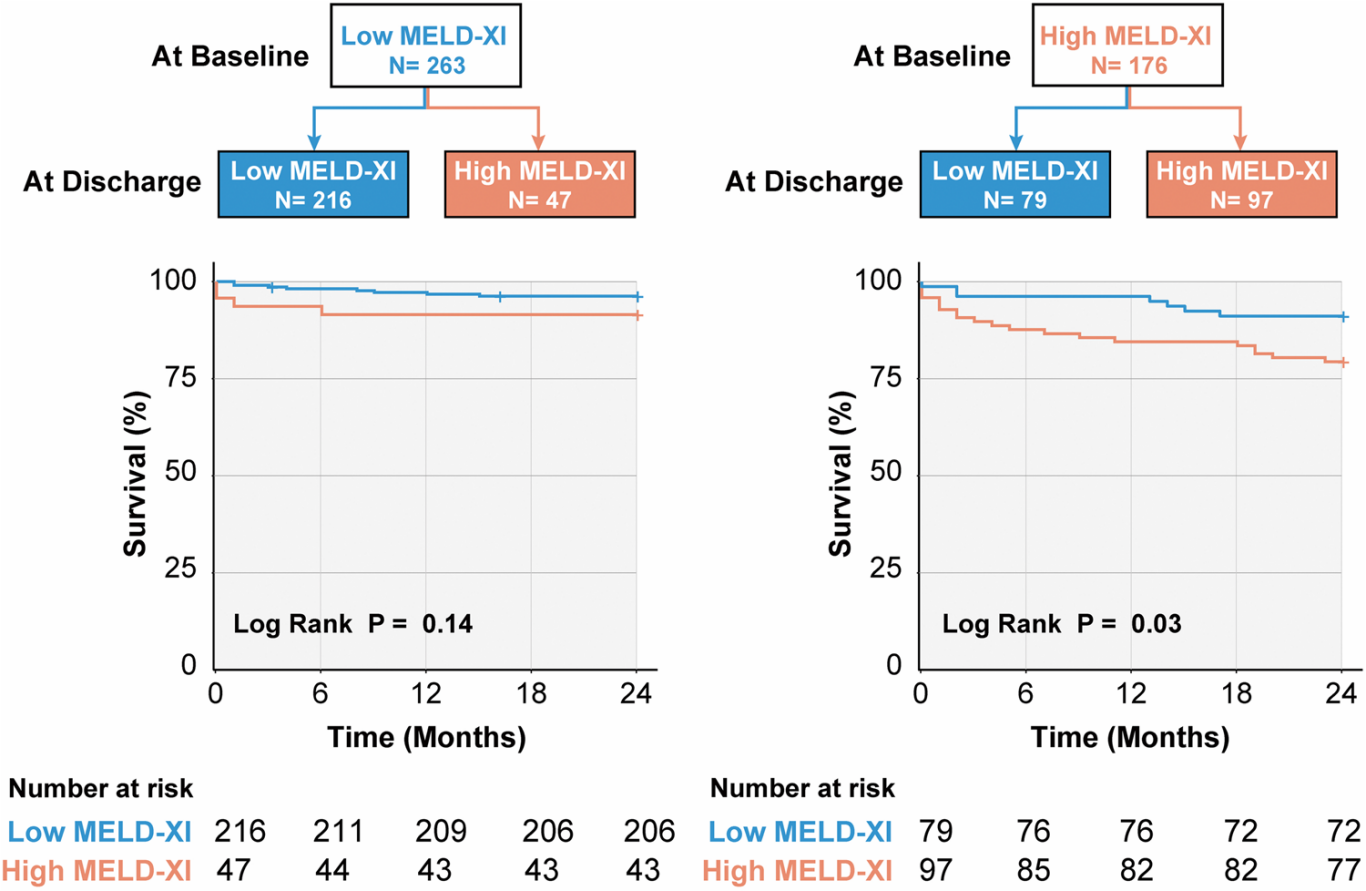
Kaplan–Meier curves for 2‐year all‐cause mortality according to the change of MELD‐XI score after transcatheter aortic valve implantation. MELD, model for end‐stage liver disease.

Among 263 patients with low MELD‐XI scores at baseline, 216 (82.1%) preserved low MELD‐XI scores at discharge, while 47 (36.1%) deteriorated into high MELD‐XI scores, and they had higher risk in peri‐TAVI mortality within 30 days (*p* = .03), but there was no significant difference of 2‐year mortality between these two groups (*p* = .14). Among 176 patients with high MELD‐XI scores at baseline, 97 (55.1%) remained with high MELD‐XI scores, and they had higher risk in 2‐year mortality than patients reversibly improved from high to low MELD‐XI scores (*p* = .03) (Figure 2). Different clinical characteristics of each group are shown in Table 3. Factors associated with reversible high MELD‐XI score were low MELD‐XI score at baseline (≤12.00, OR: 2.02 [1.04–3.94], *p* = .04), high aortic valve peak velocity (≥5 m/s, OR: 2.17 [1.11–4.24], *p* = .02), and low BMI (≤25 kg/m^2^, OR: 2.73 [1.25–5.98], *p* = .01) on multivariable analysis.

**Table 3:** Difference of clinical characteristics between different MELD‐XI change groups

A. Patients in high MELD‐XI group at baseline (*N* = 176)			
	High MELD‐XI group at discharge (*N* = 97)	Low MELD‐XI group at discharge (*N* = 79)	*p*
Age, years	73 ± 6	75 ± 6	.01
Body mass index, kg/m^2^	23.0 ± 3.9	21.8 ± 3.3	.03
Aortic valve mean gradient, mmHg	50 (44–62)	60 (49–72)	.001
Aortic valve peak velocity, m/s	4.4 (4.1–4.9)	4.8 (4.4–5.4)	.001
≥Moderate MR severity after TAVI	12 (12.4)	2 (2.6)	.02
≥Moderate TR severity after TAVI	13 (13.4)	2 (2.6)	.01
MELD‐XI score at baseline	13.66 (11.73–15.34)	12.22 (11.35–14.06)	.001
MELD‐XI score at discharge	13.11 (11.78–14.56)	9.44 (9.44–9.95)	.001

B. Patients in low MELD‐XI group at baseline (*N* = 263)			
	High MELD‐XI group at discharge (*N* = 47)	Low MELD‐XI group at discharge (*N* = 126)	*p*
STS score, %	7.3 (5.0–9.6)	6.1 (3.7–8.2)	.01
COPD	32 (68.1)	110 (50.9)	.03
≥Moderate MR severity	28 (13.1)	2 (0.4)	.02
History of atrial arrhythmia	11 (23.9)	22 (10.3)	.01

*Note*: Only variables with *p* < .05 showed in the table.

Abbreviations: COPD, chronic obstructive pulmonary disease; MELD, model for end‐stage liver disease; MR, mitral regurgitation; STS, Society of Thoracic Surgeons; TAVI,  transcatheter aortic valve implantation; TR, tricuspid regurgitation.

### 3.5 Hepatorenal function during follow‐up

A total of 359 (81.8%) patients had hepatorenal function assessments during follow‐up. Characteristics of patients with or without follow‐up hepatorenal function data showed in Supporting Information:  Table 2. The follow‐up creatinine was 0.96 mg/dl (interquartile range: 0.81–1.15 mg/dl), with a mean increase of 0.12 mg/dl compared with that at discharge (*p* < .001), but had no significant difference from that at baseline (*p* = .67). The follow‐up total bilirubin was 0.74 mg/dl (interquartile range: 0.56–0.96 mg/dl). Among 82 patients with high total bilirubin (≥1 mg/dl) at baseline, improvements were seen in 43 (52.4%) with total bilirubin decreasing to normal during follow‐up.

During follow‐up, the MELD‐XI score was 9.78 (interquartile range: 9.44–11.69). High MELD‐XI at discharge was one of the independent predictor factors of high MELD‐XI score during follow‐up (OR: 3.87 [2.21–6.78], *p* < .001) on multivariable analysis, together with high MELD‐XI at baseline (OR: 5.70 [3.33–9.77], *p* < .001), BMI (OR: 1.10 [1.01–1.19], *p* = .02), and aortic valve peak velocity (OR: 0.72 [0.53‐0.98], *p* = .03). Patients who died within 2 years after TAVI had significantly higher follow‐up MELD‐XI score (12.77 [9.44–23.29] versus 9.65 [9.44–11.35]; *p* = .003).

**Figure 3:**
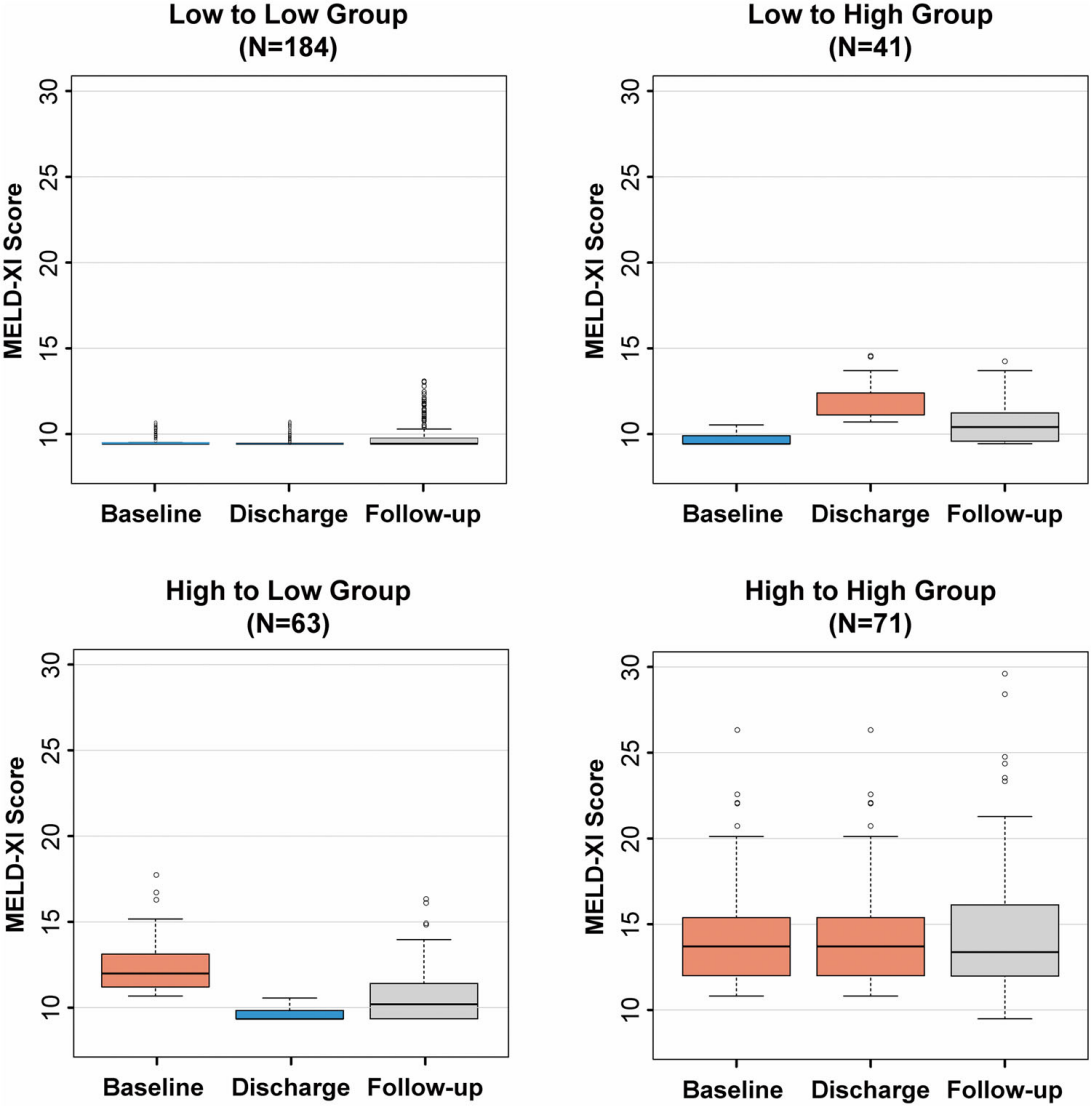
Changes of MELD‐XI score in time course. MELD, model for end‐stage liver disease.

Further analysis was performed in subgroups according to the MELD‐XI score change between baseline and discharge (Figure 3). Among 184 patients in the low MELD‐XI group at baseline and discharge, the MELD‐XI score had no significant change during follow‐up (*p* = .06). For the 41 patients whose MELD‐XI score was low at baseline but high at discharge, the MELD‐XI score during follow‐up was 10.40 (interquartile range: 9.51–11.28), and 61.0% of them restored to low MELD‐XI score group during follow‐up. Among 63 patients who improved from a high‐to‐low MELD‐XI score group, the MELD‐XI score during follow‐up was 10.28 (interquartile range: 9.44–11.54), which was significantly lower than that at baseline with a mean reduction of 1.63 (*p* < .001). Among 71 patients whose MELD‐XI scores were high at both baseline and discharge, the MELD‐XI score during follow‐up was 13.32 (interquartile range: 11.90–16.19), which had no significant difference with that at baseline (*p* = .55).

## 4 DISCUSSION

To our knowledge, this is the first study to examine the association of dynamic hepatorenal function changes with intermediate‐term mortality after TAVI and look for the factors associated with reversible hepatorenal dysfunction. Our main findings include the following: (1) both high creatinine and total bilirubin beyond normal level were associated with a significant increase in 2‐year all‐cause mortality, and the baseline high MELD‐XI score was an independent risk factor of 2‐year all‐cause mortality; (2) for patients with high MELD‐XI score at baseline, they would have higher mortality if the hepatorenal dysfunction remained irreversible high after TAVI; (3) patients with high aortic valve peak velocity but relatively low MELD‐XI score were more likely to derive hepatorenal function improvement from TAVI, while patients with high BMI were not; and (4) for patients with reversible hepatorenal dysfunction, the benefit was maintained during follow‐up.

Arai et al.^23^ used the MELD‐XI score to examine hepatorenal dysfunction in 749 patients undergoing TAVI between 2013 and 2015 in Japan, and they found that a higher MELD‐XI score at baseline was associated with higher 30‐day mortality and was an independent predictor of 6‐month mortality. We found that a high MELD‐XI score at baseline was still an independent predictor for 2‐year mortality after TAVI, even excluding the influence of peri‐operative deaths within 30 days. It should also be mentioned that our data showed that aortic regurgitation before TAVI was associated with a significantly better 2‐year outcome after multivariate adjustment, which seemed to be counterintuitive, but it was consistent with previous study.^24^, ^25^

The STS score, which is one of the risk assessment tools for mortality prediction in patients undergoing cardiovascular treatments, involves creatinine as a marker of renal function, but it does not quantify liver dysfunction severity.^26^ When combining the STS score with baseline liver dysfunction severity reflected by total bilirubin, it showed better performance in both peri‐TAVI and intermediate‐term mortality prediction. It suggested that hepatorenal function at baseline is also helpful in pre‐TAVI evaluation.

In patients with hepatorenal dysfunction at baseline, those with high aortic valve peak velocity (≥5 m/s) but relatively low MELD‐XI score (higher than 10.70 but not higher than 12.00) derived the most benefit from TAVI by possessing reversible hepatorenal function improvement. It can be inferred that, the more severe the stenosis, the more its role matters in hepatorenal dysfunction. Thus, the management directed toward relieving aortic valve obstruction can achieve a more significant effect on hepatorenal function recovery. Meanwhile, patients with relatively lower MELD‐XI scores have a better ability to resilience in hepatorenal function, which is similar to the result of a study by Witberg et al.^6^

Although previous studies showed that overweight and obese patients with established cardiovascular diseases tend to have a lower incidence of all‐cause death,^27^ the impacts of high BMI on complications of TAVI were not always positive,^28^ and prior studies also found that overweight and obesity were risk factors for chronic kidney disease.^29^ This study showed that high BMI (≥25 kg/m^2^) was one of the risk factors for irreversible hepatorenal dysfunction. It suggested that the appropriate BMI, which allowed TAVI patients to have better outcomes and quality of lives remained to be further analyzed.

## 5 LIMITATION

This study has several limitations. First, as it is a retrospective study, the association between hepatorenal function and mortality could have been influenced by other potential confounders that were not controlled for in the regression models. Second, we analyzed the changes in the patients' latest MELD‐XI scores within 2 years after TAVI, which may be biased due to the deaths of patients during this period. But for patients who died within 2 years but not within 30 days, the majority of them still had the latest hepatorenal function tests before death, and we involved the data in the analysis. Third, obesity (BMI ≥ 25 kg/m^2^) was one of the risk factors for irreversible hepatorenal dysfunction after TAVI, but given the relatively low BMI of Asians, obese patients may be underrepresented in our cohorts.

## 6 CONCLUSION

In conclusion, we found that hepatorenal function assessed by MELD‐XI score before TAVI was independently associated with 2‐year all‐cause mortality. TAVI had a long‐standing benefit in the improvement of hepatorenal function in patients with reversible hepatorenal dysfunction, and these patients had a lower risk of mortality. For patients with irreversible hepatorenal dysfunction after TAVI, further optimization of the subsequent treatments after TAVI is needed to improve the outcomes.

## CONFLICT OF INTEREST

The authors declare no conflict of interest.

## Supporting information

Supplementary information.

## Data Availability

The data underlying this article will be shared on reasonable request to the corresponding author.
