# Supplementary material for: Prognostic value of the dynamic hepatorenal function on intermediate‐term mortality in TAVI patients with survival to discharge
Source: Clin Cardiol. 2022 Nov 30;46(1):84–91. doi: 10.1002/clc.23940 (PMC9849429; doi:10.1002/clc.23940)
Supplement: Supplementary file 1 — Supplementary information. [file CLC-46-84-s001.docx]

**SUPPLEMENTAL MATERIAL.**

Supplemental Figures 1–3

Supplemental Tables 1–2

**
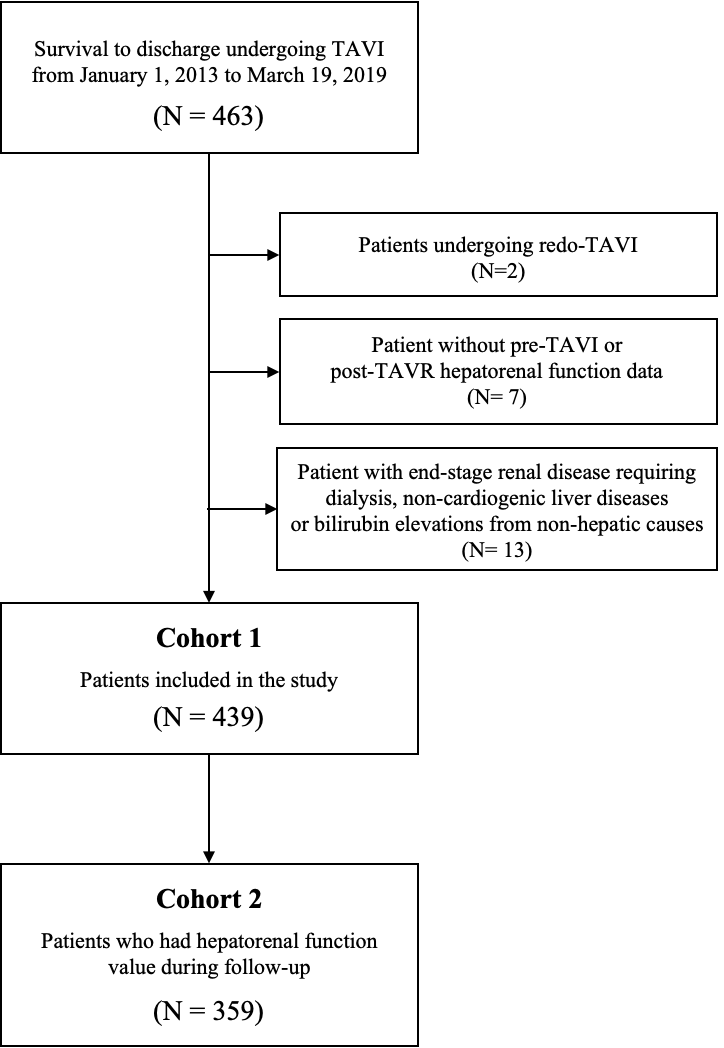
**

**Supplemental Figure 1.** Flow chart of patient selection.

TAVI = transcatheter aortic valve implantation


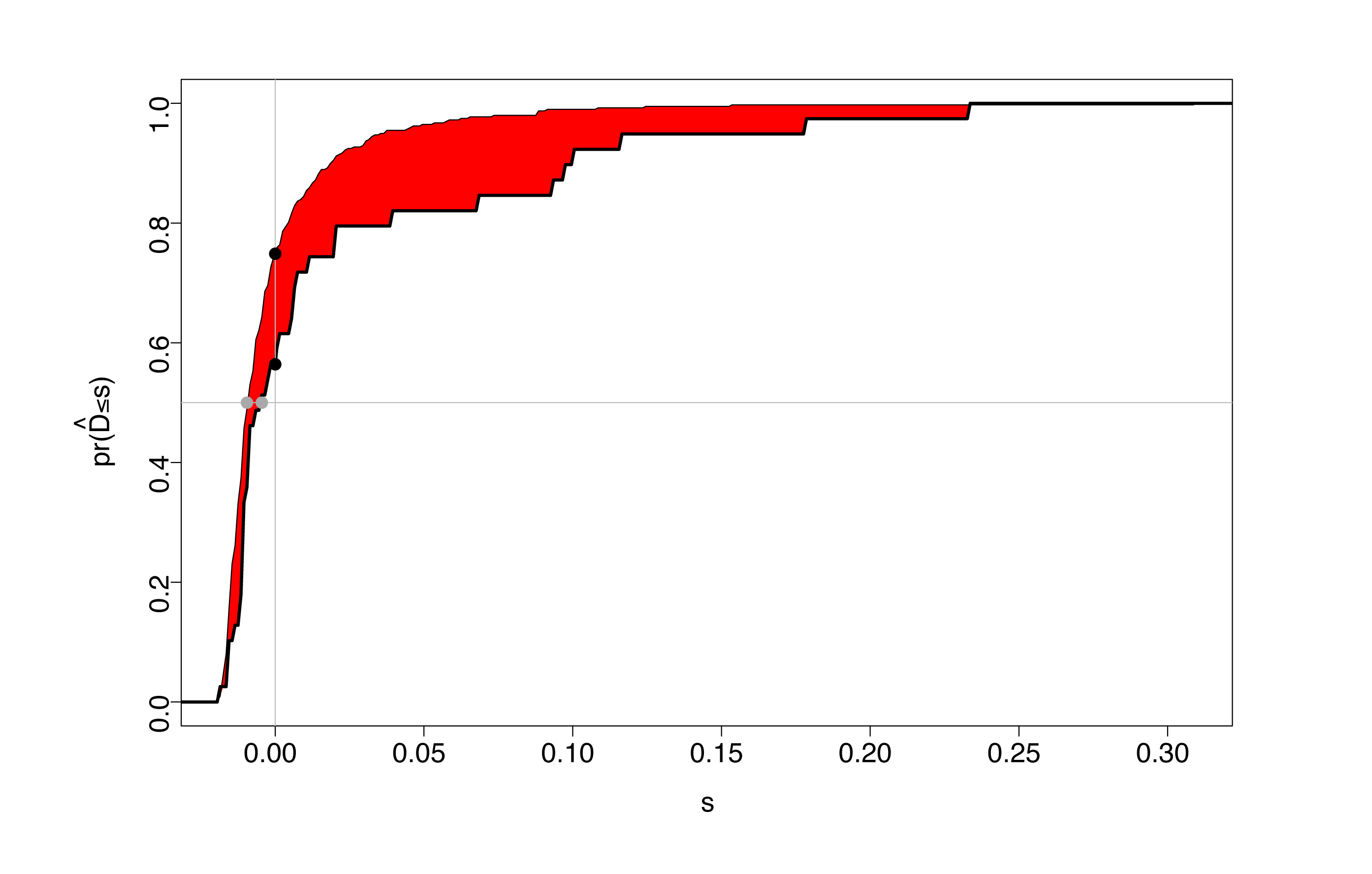


**Supplemental Figure 2.** Integrated Discrimination Improvement of MELD-XI score compared with CRE in prediction of 2-year mortality.

**
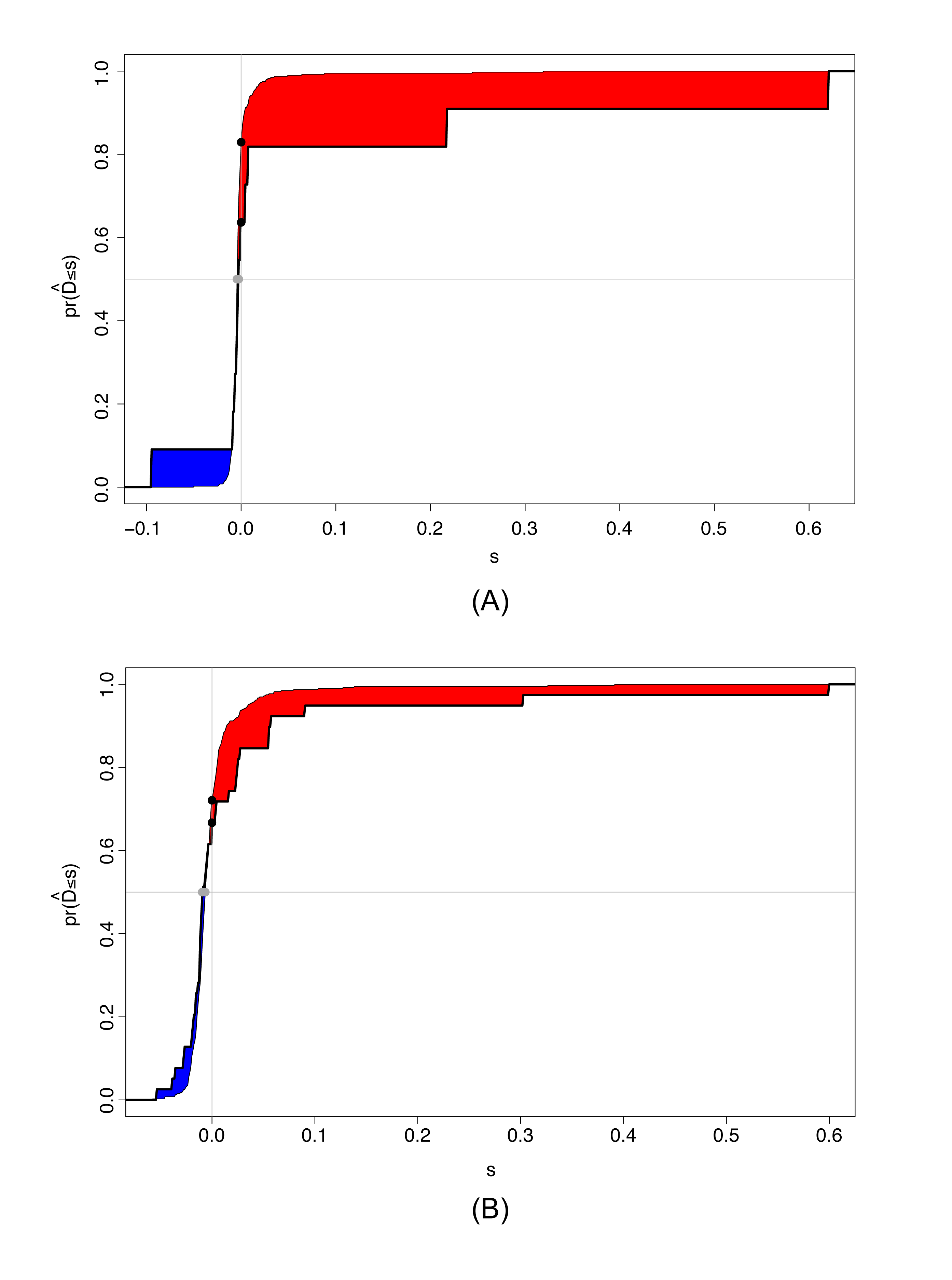
**

**Supplemental Figure 3. (A)** Integrated Discrimination Improvement of STS score combined with total bilirubin compared with STS score in prediction of 30-day mortality. **(B)** Integrated Discrimination Improvement of STS score combined with total bilirubin compared with STS score in prediction of 2-year mortality.

**Supplemental Table 1.** Multivariate model showing risk factors for hepatorenal dysfunction (MELD-XI score) at baseline.

|  | b Coefficient ± SE | p |
| --- | --- | --- |
| ≥ Moderate tricuspid regurgitation | 1.64±0.36 | <0.001 |
| ≥ Moderate mitral regurgitation | 1.04±0.36 | 0.004 |
| Diabetes mellitus | 0.65±0.32 | 0.04 |
| Male | 0.64±0.25 | 0.01 |
| Aortic valve peak velocity, per 1 m/s | -0.44±0.14 | 0.002 |
| STS score, % | 0.20±0.03 | <0.001 |

MELD = model for end-stage liver disease; STS = Society of Thoracic Surgeons.

**Supplemental Table 2.** Characteristics of patients with or without follow-up hepatorenal function data.

|  | follow-up group  (n=362) | without  follow-up group  (n=81) | p value |
| --- | --- | --- | --- |
| Demographics |  |  |  |
| Age, yrs | 74±7 | 74±5 | 0.69 |
| Male | 198(54.7) | 50(61.7) | 0.25 |
| Body mass index, kg/m2 | 22.3±3.5 | 22.9±4.3 | 0.26 |
| STS score, % | 7.1(4.5-8.9) | 8.2(5.7-11.9) | 0.01 |
| STS score ≥8% | 143(39.5) | 44(54.3) | 0.02 |
| Symptoms |  |  |  |
| Syncope | 44(12.2) | 8(9.9) | 0.73 |
| Chest pain | 104(28.7) | 23(28.4) | 0.71 |
| Dyspnea | 331(91.4) | 68(84.0) | 0.99 |
| NYHA III or IV | 315(87.0) | 75(92.6) | 0.11 |
| Cardiovascular conditions |  |  |  |
| Hypertension | 154(42.6) | 37(45.7) | 0.64 |
| History of atrial arrhythmia | 55(15.2) | 16(19.8) | 0.22 |
| Coronary artery disease | 137(37.8) | 33(40.7) | 0.31 |
| Previous MI | 4(1.1) | 4(4.9) | 0.13 |
| Previous PCI | 29(10.0) | 11(13.6) | 0.20 |
| PCI during TAVI | 3(0.8) | 1(1.2) | 0.73 |
| Peripheral arterial disease | 169(46.7) | 34(42.0) | 0.88 |
| Prior stroke or TIA | 80(22.1) | 17(21.0) | 0.89 |
| Noncardiac conditions |  |  |  |
| COPD | 209(57.7) | 50(61.7) | 0.51 |
| Diabetes mellitus | 65(18) | 19(23.5) | 0.29 |
| Echocardiography |  |  |  |
| LVEF, % | 56±14 | 52±16 | 0.02 |
| LVEF <35% | 38(10.5) | 17(21.5) | 0.03 |
| Aortic valve mean gradient, mmHg | 59(47-72) | 60(48-74) | 0.97 |
| Aortic valve peak velocity, m/s | 4.8(4.3-5.4) | 5.0(4.3-5.5) | 0.73 |
| ≥Moderate AR severity | 110(30.4) | 19(23.5) | 0.40 |
| ≥Moderate MR severity | 55(15.2) | 8(9.9) | 0.28 |
| ≥ Moderate TR severity | 48(13.3) | 16(19.8) | 0.11 |
| IVS | 13.8±2.3 | 13.6±2.2 | 0.49 |
| Procedural and postprocedural data |  |  |  |
| Self-expandable valve | 338(93.4) | 79(97.5) | 0.06 |
| Aortic valve mean gradient after TAVI, mmHg | 11(8-16) | 12(9-16) | 0.86 |
| Aortic valve peak velocity after TAVI, m/s | 2.3(2.0-2.6) | 2.3(2.1-2.6) | 0.70 |
| ≥Moderate AR severity after TAVI | 5(1.4) | 0(0) | 0.03 |
| ≥Moderate MR severity after TAVI | 21(5.8) | 4(4.9) | 0.81 |
| ≥Moderate TR severity after TAVI | 20(5.5) | 7(8.6) | 0.36 |
| LVEF after TAVI, % | 57±12 | 54±13 | 0.04 |
| LVEF after TAVI <35% | 24(6.6) | 9(11.1) | 0.23 |
| Length of admission, d | 9(7-10) | 9(7-12) | 0.41 |
| Year of procedure |  |  |  |
| 2013-2016 | 129(35.6) | 49(60.5) | <0.001 |
| 2017-2019 | 233(64.4) | 32(39.5) | <0.001 |
| MELD-XI score at baseline | 9.91(9.44-11.93) | 11.09(9.44-13.90) | 0.03 |
| High MELD-XI at baseline | 137(37.8) | 43(53.1) | 0.01 |
| MELD-XI score at discharge | 9.44(9.44-11.25) | 9.84(9.44-13.12) | 0.03 |
| High MELD-XI at discharge | 52(14.4) | 24(29.6) | 0.01 |

Values are mean ± SD or n (%).

AR = aortic regurgitation; COPD = chronic obstructive pulmonary disease; LVEF = left ventricular ejection fractions; MI = myocardial infarction; MR = mitral regurgitation; NYHA = New York Heart Association; PCI = percutaneous coronary intervention; STS = Society of Thoracic Surgeons; TAVI = transcatheter aortic valve implantation; TIA = transient ischemia attack; TR = tricuspid regurgitation.
